# An Unprecedented alteration in mode of action of IsCT resulting its translocation into bacterial cytoplasm and inhibition of macromolecular syntheses

**DOI:** 10.1038/srep09127

**Published:** 2015-03-16

**Authors:** Jitendra K. Tripathi, Manoj Kathuria, Amit Kumar, Kalyan Mitra, Jimut K. Ghosh

**Affiliations:** 1 Molecular and Structural Biology Division, CSIR-Central Drug Research Institute, Sector 10, Jankipuram Extension, Sitapur Road, Lucknow–226 031, India; 2 Electron Microscopy Unit, CSIR-Central Drug Research Institute, Sector 10, Jankipuram Extension, Sitapur Road, Lucknow–226 031, India

## Abstract

IsCT, a 13-residue, non-cell-selective antimicrobial peptide is comprised of mostly hydrophobic residues and lesser cationic residues. Assuming that placement of an additional positive charge in the non-polar face of IsCT could reduce its hydrophobic interaction, resulting in its reduction of cytotoxicity, an analog, I9K-IsCT was designed. Two more analogs, namely, E7K-IsCT and E7K,I9K-IsCT, were designed to investigate the impact of positive charges in the polar face as well as polar and non-polar faces at a time. These amino acid substitutions resulted in a significant enhancement of therapeutic potential of IsCT. IsCT and E7K-IsCT seem to target bacterial membrane for their anti-bacterial activity. However, I9K-IsCT and E7K,I9K-IsCT inhibited nucleic acid and protein syntheses in tested *E. coli* without perturbing its membrane. This was further supported by the observation that NBD-IsCT localized onto bacterial membrane while NBD-labeled I9K-IsCT and E7K,I9K-IsCT translocated into bacterial cytoplasm. Interestingly, IsCT and E7K-IsCT were significantly helical while I9K-IsCT and E7K,I9K-IsCT were mostly unstructured with no helix content in presence of mammalian and bacterial membrane-mimetic lipid vesicles. Altogether, the results identify two novel cell-selective analogs of IsCT with new prototype amino acid sequences that can translocate into bacterial cytoplasm without any helical structure and inhibit macromolecular syntheses.

Antimicrobial peptides (AMPs) are small cationic amphiphilic peptides found in almost all living organisms that play a key role in the host defense mechanism against microbial infections. In comparison to conventional antibiotics, which target specific metabolic processes, majority of the AMPs adopt globally amphipathic conformations and kill bacteria through disruption of their membranes^1^,^2^,^3^,^4^,^5^,^6^,^7^. However, the number of AMPs targeting other than bacterial membranes is relatively much smaller though it is increasing^8^,^9^,^10^,^11^,^12^,^13^,^14^,^15^,^16^,^17^,^18^,^19^,^20^,^21^. Looking at their broad spectrum activity, antimicrobial peptides are considered as lead molecules for the development of new antimicrobial agents. However, developing new antimicrobial agents with selective lytic activity only towards microorganisms is a major challenge, especially when the target is cell membrane which is somewhat non-specific. To become a candidate for the development of new antimicrobial drug, an antimicrobial peptide with intracellular target needs to inhibit the intracellular events only in microorganisms but not in normal human cells. Therefore, the design of antimicrobial peptides with selective activity either to microbial membrane or to the intracellular mechanism of the microorganisms is a crucial step towards the development of potentially new antimicrobial agents.

IsCT is one of the shortest natural cytotoxic peptides consisting of only 13 amino acid residues with α-helical secondary structure and antimicrobial property which has been isolated from the scorpion, *Opisthacanthus madagascariensis*^22^. IsCT also shows potent cytolytic activity against mammalian cells^23^. Since a short linear antimicrobial peptide with selective activity to microorganisms would be an attractive candidate as a lead therapeutic agent, we aimed at that with IsCT. IsCT comprises of mostly hydrophobic residues with relatively less number of cationic residues (net charge, +1). We identified a leucine zipper like sequence in IsCT which was not reported before. The hydrophobic leucine/isoleucine residues at ‘a’ and/or ‘d’ positions of a leucine zipper like sequence have been shown to play a crucial role in cytotoxicity of other antimicrobial peptides^1^,^24^. Substitution of amino acids at ‘a’ and/or ‘d’ positions of leucine zipper like sequences of these antimicrobial peptides with alanine residues drastically reduced their cytotoxicity probably as a result of decrease in peptide-induced permeabilization of mammalian cell membrane^1^,^2^,^5^,^24^. Interestingly, the alanine-substituted analogs of these antimicrobial peptides retained their antimicrobial as well as membrane permeabilization property towards bacterial membrane or bacterial membrane mimetic, negatively charged lipid vesicles. A different approach has been adopted for the design of cell-selective analogs of IsCT. We wanted to introduce an additional positive charge in the hydrophobic face of IsCT assuming that it could impair the hydrophobic interaction in the molecule further resulting in its decrease of cytotoxicity. We also intended to incorporate a substitution in place of the isoleucine residue at 9^th^ position which is an ‘a’ position of the identified leucine zipper sequence of IsCT since such substitution often results in the reduced cytotoxicity of the peptide as already mentioned. Thus the isoleucine residue at 9^th^ position which is also located in the hydrophobic face of IsCT as per the helical wheel was substituted with a lysine residue. To look into the influence of additional positive charge in the polar face of the molecule, a glutamic acid residue at 7^th^ position was replaced by lysine residue. This analog was reported some time ago by others whose antimicrobial and cytotoxic properties were only studied^22^. An analog of IsCT was also designed to study the impact of introduction of positive charges in both polar and non-polar face of the molecule by replacing the glutamic acid and isoleucine residues at 7^th^ and 9^th^ positions with two lysine residues. Detailed structural, functional and biological properties of the native peptide and its analogs were carried out which revealed the design of novel analogs of IsCT that selectively translocated into bacterial cytoplasm and impaired its biomolecular syntheses without causing any damage to the membrane.

## Results

### Design of IsCT analogs

As already described in the last part of introduction section along with the rationale that three analogs of IsCT, namely I9K-IsCT, E7K-IsCT and E7K,I9K-IsCT were designed. Table 1 shows the amino acid sequence and designation of the peptides; supplementary Fig. S1 shows helical wheel projections of IsCT and their analogues and calculated physicochemical parameters of these peptides are shown in supplementary Table S1. Data in supplementary Table S1 clearly indicate a significant reduction in hydrophobic character of IsCT and E7K-IsCT following the substitution of isoleucine at 9^th^ position with a lysine residue in these peptides.

### Isoleucine to lysine substitution at 9^th^ position drastically reduced the cytotoxicity of IsCT

**Figure 1:**
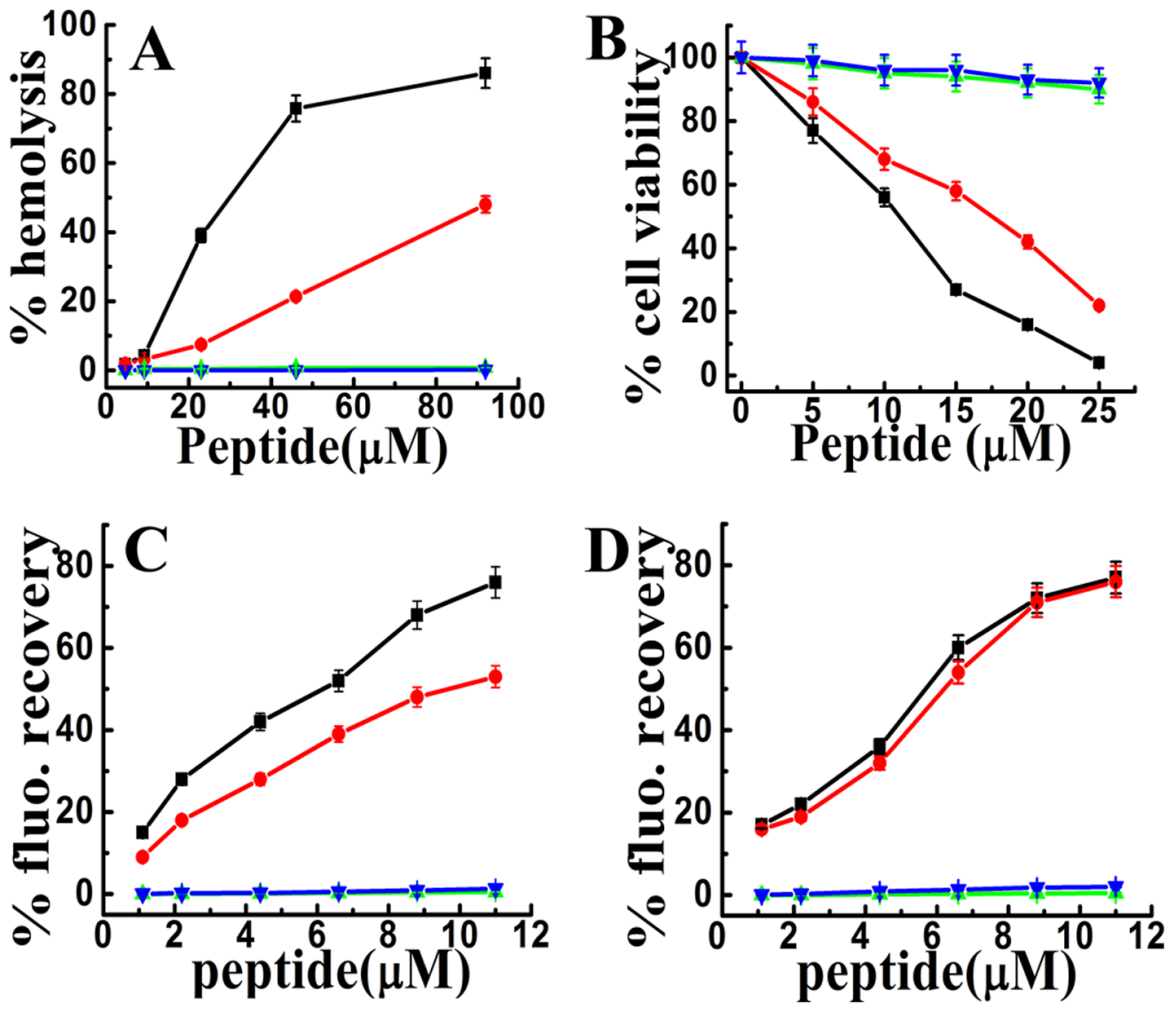
Determination of cytotoxicity of IsCT and its analogs and permeabilization of different kinds of lipid vesicles in the presence of these peptides. (A) and (B) show the dose-dependent hemolysis of hRBCs and viability of murine 3T3 cells respectively in the presence of IsCT, E7K-IsCT, I9K-IsCT and E7K,I9K-IsCT. (C) and (D) show the plot of fluorescence recovery which is a measure of peptide-induced membrane permeabilization vs peptide concentration (μM) in mammalian and bacterial membrane mimetic PC/Chol (8:1, w/w) and PC/PG (3:1, w/w) lipid vesicles respectively. Symbols: black square, IsCT, red circle, E7K-IsCT; green upright triangle, I9K-IsCT and blue inverted triangle, E7K,I9K-IsCT. Each data point is an average of three independent experiments and error bar represents the standard deviation.

Cytotoxicity of IsCT and its designed analogs was examined by assaying the peptides-induced lyses of human red blood cells (hRBCs) and the viability of murine 3T3 cells in the presence of these peptides by MTT assay. The native IsCT showed significant lysis of hRBCs and its analog E7K-IsCT showed ~50% hemolytic activity of the native peptide as reported earlier^18^. However, the introduction of lysine residue instead of isoleucine at 9^th^ position of the peptide drastically reduced the hemolytic activity of IsCT (Fig. 1A). The analog with substitutions of lysine residues at 7^th^ and 9^th^ positions was extremely non-toxic. At 90 μM peptide concentration IsCT, E7K-IsCT, I9K-IsCT and E7K,I9K-IsCT showed ~86, 48, 0.4 and 0.14 percent of lysis of hRBCs respectively.

Viability of 3T3 cells as determined by MTT assay in the presence of these peptides follow the same trend as their hemolytic activity data (Fig. 1B). The results altogether indicate a significant effect of substitution of isoleucine residue at 9^th^ position which is also an ‘a’ position in the leucine zipper sequence of the peptide on the cytotoxicity of IsCT.

### The designed analogs showed either similar or higher antibacterial property than IsCT

The native IsCT and its analogs were examined for bacterial growth-inhibiting activity in liquid culture against Gram-positive and Gram-negative bacteria. In contrast to their cytotoxicity, the IsCT-analogs exhibited appreciable antimicrobial activities. IsCT showed substantial antibacterial activity; E7K-IsCT showed ~30% lower MIC values against the bacteria as compared to that of the native peptide indicating its higher activity which supports a previous report^22^. The activity of I9K-IsCT was slightly lower than IsCT. However, the analog, E7K,I9K-IsCT showed ~2–3 fold higher activity than native IsCT (Table 2) against the tested bacteria. The results suggest that the introduction of positive charge on the polar and non-polar face of IsCT could have a synergistic effect on its antibacterial property.

Therapeutic index of the peptides is calculated by the ratio of HC_50_ (mean concentration of peptide producing 50% hemolysis) and MIC (minimum inhibitory concentration of peptide, related to its antimicrobial activity)^25^; thus, the larger value in therapeutic index indicates greater antimicrobial specificity. As shown in Table 3, therapeutic index for IsCT wild, E7K-IsCT, I9K-IsCT and E7K,I9K-IsCT are 4.83, 15.34, 41.60 and 166.11 respectively.

### Differences among IsCT and its analogs in peptides-induced permeabilization of zwitterionic and negatively charged lipid vesicles

Since majority of the naturally occurring antimicrobial peptides and their analogs are membrane-active, to understand the basis of cytotoxic and antibacterial activities of IsCT and its analogs, peptides-induced permeabilization of mammalian membrane-mimetic, PC/Chol (phosphatidylcholine/Cholesterol) and bacterial membrane-mimetic, PC/PG (phosphatidylcholine/phosphatidylglycerol) lipid vesicles was studied. Consistent with cytotoxic activity, IsCT induced the maximum permeabilization (expressed as the percentage of fluorescence recovery) in PC/Chol lipid vesicles followed by E7K-IsCT (Fig. 1C and D). I9K-IsCT and E7K,I9K-IsCT induced almost no permeabilization of PC/Chol lipid vesicles (Fig. 1C) which indicates the negligible efficacy of these IsCT-analogs to permeabilize the mammalian cell membrane mimetic lipid vesicles and thus show negligible cytoxicity. Interestingly, though IsCT and E7K-IsCT permeabilized bacterial membrane mimetic PC/PG lipid vesicles, despite significant antibacterial properties I9K-IsCT and E7K,I9K-IsCT failed to permeabilize the same kind of lipid vesicles. On the basis of the data, we anticipated that though the native IsCT and its analog, E7K-IsCT are membrane-interacting molecules, probably I9K-IsCT and E7K,I9K-IsCT do not target bacterial cell membrane to exhibit their antibacterial properties.

### In Contrast to IsCT and E7K-IsCT, I9K-IsCT and E7K,I9K-IsCT did not damage the bacterial and hRBCs membrane

**Figure 2:**
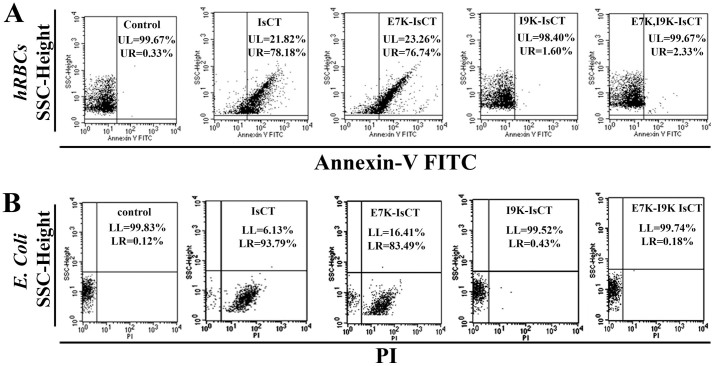
(A) Peptides induced damage of membrane organization of hRBCs as detected by FITC-annexinV staining following the treatment of peptides. Upper left quadrant of each panel depicts unstained cells, whereas the upper right quadrant depicts the stained cells. Concentrations of the peptides were ~30.0 μM. (B) Peptides induced membrane damage of *E. coli* ATCC25922 as detected by PI staining. Lower left quadrant of each panel depicts unstained cells whereas the lower right quadrant depicts the stained cells. Concentrations of the peptides were ~5.0 μM. 10000 events were recorded for each of the experiments.

To further look into the mechanism of action of these peptides, FITC-annexin V staining of hRBCs was studied following their treatment with IsCT and its analogs. IsCT and E7K-IsCT extensively damaged to hRBCs membrane while I9K-IsCT and E7K,I9K-IsCT were almost inactive as evidenced by the characteristic staining of hRBCs with FITC-annnexin V. The data are supportive of the relative cytotoxic properties of IsCT and analogs (Fig. 2A).

The peptide-induced damage of bacterial membrane was studied by propidium iodide (PI) staining of *E. coli* (ATCC25922) following the treatment with IsCT and its analogs. This dye binds to nucleic acids, which is possible only after the damage of bacterial membrane. IsCT and E7K-IsCT induced extensive PI staining of *E. coli* (Fig. 2B) indicating their bacterial membrane damaging property, whereas I9K-IsCT and E7K,I9K-IsCT induced insignificant damage to bacterial membrane as evident from the insignificant PI staining of bacteria after the treatment with these two peptides. The results clearly suggested that unlike IsCT and E7K-IsCT, the other two analogs, I9K-IsCT and E7K,I9K-IsCT did not show the ability to damage the bacterial membrane.

### Significant differences among IsCT and its analogs in localization onto zwitterionic and negatively charged lipid vesicles

**Figure 3:**
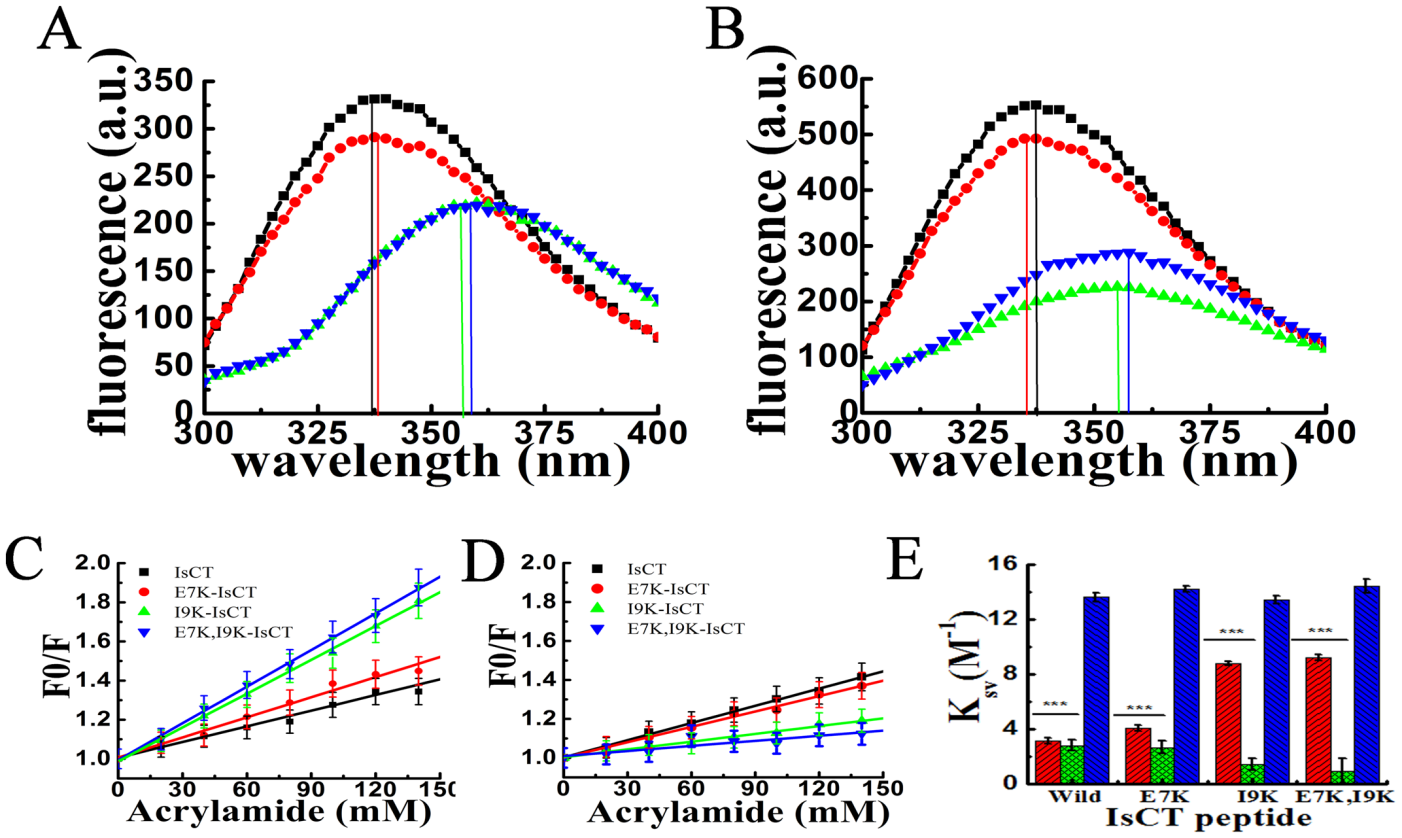
Determination of environment of the tryptophan residues of IsCT and its analogs in presence of PC/Chol (A) and PC/PG (B) lipid vesicles. Each of the Peptides (~2.0 μM) was added to PBS with subsequent addition of ~450 μM of either of the lipid vesicles. Symbols: square, IsCT wild; circle, E7K-IsCT; up-right triangle, I9K-IsCT; and inverted triangle, E7K,I9K-IsCT. (C) and (D) depict the Stern-Volmer plots for the acrylamide quenching of tryptophan fluorescence of the peptides as designated in the plots in presence of PC/Chol (C) and PC/PG (D) lipid vesicles whereas panel, (E) shows the corresponding Stern-Volmer constants (*K_SV_*) for each of the peptides in different environments as indicated in the plot. Symbols: black square, IsCT; red circle, E7K-IsCT; green up-right triangle, I9K-IsCT; and blue inverted triangle, E7K,I9K-IsCT. In Fig. 3E the results are presented as means ± SD; n = 3. ***, P < 0.001 versus buffer alone.

The sensitivity of the fluorescence emission of the tryptophan residue on its environment allows us to monitor the binding/localizations of these peptides having tryptophan residue onto the phospholipid membrane. All four peptides exhibited an emission maximum at ~357 nm in PBS, indicating the location of their tryptophan residues in a polar environment (supplementary Fig. S2A). However, in presence of mammalian membrane mimetic, PC/Chol lipid vesicles, a large blue shift (~20 nm) was observed for IsCT and E7K-IsCT. On the other hand, tryptophan emission maxima of I9K-IsCT and E7K,I9K-IsCT showed a negligible blue shift in the same environment (Fig. 3A). A similar result was observed in the presence of bacterial membrane mimetic, PC/PG lipid vesicles (Fig. 3B). The results suggested that probably the tryptophan residues of IsCT and E7K-IsCT were localized toward the hydrophobic core region of both zwitterionic and negatively charged phospholipid lipid bilayer while the tryptophan residues of I9K-IsCT and E7K,I9K-IsCT were located more towards the polar milieu of these lipid vesicles. Profiles of only PC/Chol and PC/PG lipid vesicles are shown in supplementary Fig. S2B.

### Acrylamide quenching of tryptophan fluorescence of the peptides in zwitterionic and negatively charged lipid vesicles yields different results

To further investigate on the localization of these peptides onto the mammalian and bacterial membrane mimetic environments, quenching of tryptophan fluorescence of the peptides by acrylamide was studied in the presence of zwitterionic PC/Chol and negatively charged, PC/PG lipid vesicles respectively. Tryptophan fluorescence of both IsCT and E7K-IsCT showed much lesser quenching by acrylamide as compared to that of I9K-IsCT and E7K,I9K-IsCT when the peptides were bound to PC/Chol, lipid vesicles (Fig. 3C). The results indicate that tryptophan residues of IsCT and E7K-IsCT were located more towards the bilayer of PC/Chol vesicles and therefore not accessible by acrylamide, whilst tryptophan residues of I9K-IsCT and E7K,I9K-IsCT were possibly located toward the surface of zwitterionic membrane, and thus accessible to acrylamide resulting in significant quenching of their fluorescence (Fig. 3C). Though tryptophan fluorescence of all four peptides showed appreciably less acrylamide quenching in PC/PG lipid vesicles, it is noteworthy that the observed acrylamide quenching for I9K-IsCT and E7K,I9K-IsCT was even lesser than that of IsCT and E7K-IsCT (Fig. 3D). The data are indicative of relatively higher non-accessibility of acrylamide to the tryptophan residues of I9K-IsCT and E7K,I9K-IsCT in PC/PG lipid vesicles which was apparently contrary to their emission maxima at longer wavelength (Fig. 3B). Stern-Volmer constants (*Ksv*) for IsCT and its analogs in PC/PG lipid vesicles match with the relative quenching of their tryptophan fluorescence by acrylamide as described before (Fig. 3D). However, *Ksv* values of I9K-IsCT and E7K,I9K-IsCT were found significantly higher than that of IsCT and E7K-IsCT in PC/Chol lipid vesicles suggesting the higher quenching of their tryptophan fluorescence by acrylamide. *Ksv* values for all these peptides were appreciably higher in aqueous buffer indicating strong acrylamide quenching of their tryptophan fluorescence (Fig. 3E).

### Unlike IsCT and E7K-IsCT; I9K-IsCT and E7K,I9K-IsCT did not adopt appreciable helical structure in zwitterionic or negatively charged lipid vesicles

**Figure 4:**
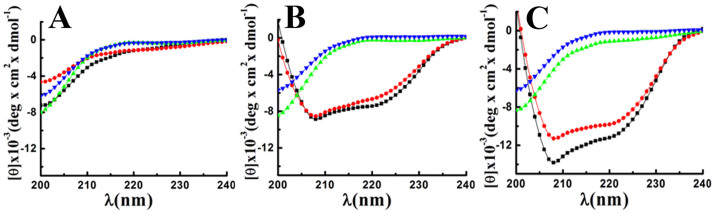
Determination of secondary structures of the peptides (~43 μM) in PBS, pH 7.4 (A); PC/Chol (B) and PC/PG (C) lipid vesicles of ~500 μM in each case. Symbols: black squares, IsCT, red circles, E7K-IsCT, green up-triangle, I9K-IsCT and blue inverted triangle, E7K,I9K-IsCT.

Secondary structures of the peptides were determined in PBS (pH 7.4), PC/Chol, (8:1 w/w) and PC/PG (3:1 w/w) lipid vesicles by circular dichroism studies and were analyzed by software J-815 SSE as shown in supplementary Table S2. None of the above mentioned peptide adopted helical structure in PBS (Fig. 4A and supplementary Table S2). IsCT and E7K-IsCT adopted appreciable helical structure in the presence of PC/Chol and PC/PG lipid vesicles (Fig. 4B, 4C and supplementary Table S2). In contrast, I9K-IsCT and E7K,I9K-IsCT did not adopt any helical structure either in the presence of PC/Chol or PC/PG lipid vesicles (Fig. 4B,4C and supplementary Table S2). As per the secondary structure analyses, major components of structures of both I9K-IsCT and E7K,I9K-IsCT are random coil. The results indicate a dramatic effect of substitution of isoleucine to lysine residue at 9^th^ position which is also an ‘a’ position of the identified leucine zipper sequence of IsCT on its secondary structure. I9K-IsCT and E7K,I9K-IsCT showed mostly random coil structures (profiles not shown) even in SDS micelle which is also employed as a bacterial membrane mimetic environment. Change in HT voltage with respect to wavelength for PBS alone as well as IsCT and its analogs in PBS are shown in supplementary Fig. S3 to show that no abrupt change in HT voltage took place while recording the CD spectra.

### Visualization of bacterial morphology after the treatments of IsCT and its analogs under the scanning electron microscope

**Figure 5:**
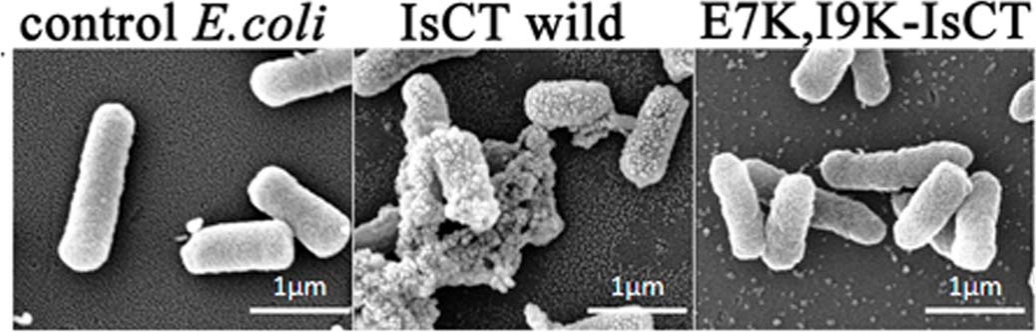
Scanning Electron Microscopy of *E. coli* ATCC 25922. Image of bacteria without any peptide treatment or with treatment of IsCT and E7K,I9K-IsCT at 10 folds of MIC as marked above the images.

To get more insight on the mode of action of IsCT and its analogs, morphology of *E*. *coli* 25922 was visualized by a scanning electron microscope (SEM) following the treatment with IsCT and one of its analogs, E7K,I9K-IsCT (as a representative) that do not permeabilize the phospholipid membrane. Untreated bacteria appeared as smooth surface under SEM (Fig. 5). *E. coli* treated with IsCT at 10 fold of MIC value for 60 min showed distinct wrinkling, surface roughening and blebbing of the membrane indicating the loss of membrane integrity (Fig. 5). In contrast, *E. coli* treated with E7K,I9K-IsCT peptide at 10 fold of MIC value for 60 min showed fairly normal and smooth surface under the SEM (Fig. 5).

### Localization of IsCT and its analogs onto bacteria by confocal microscopy

**Figure 6:**
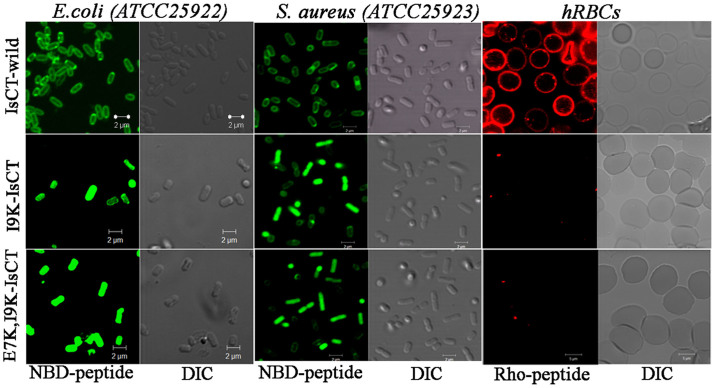
Confocal laser scanning Electron microscopic study of localization of NBD or Rho-labeled IsCT, I9K-IsCT and E7K,I9K-IsCT peptides onto *E. coli* ATCC 25922, *S. aureus* ATCC 25923 and human RBCs. Images showing the fluorescence and DIC images for the treatment of a particular peptide as marked on the images.

The cellular localization of IsCT, E7K,I9K-IsCT and I9K-IsCT onto *E. coli ATCC25922 and S. aureus ATCC25923* was studied by confocal scanning laser microscopy by employing their NBD-labeled versions. As evident from the confocal microscopic images, NBD-labeled IsCT mostly accumulated onto the bacterial membrane. On the other hand, the two IsCT analogs, I9K-IsCT and E7K,I9K-IsCT that did not permeabilize phospholipid vesicles or damage to bacterial membrane as observed previously (Figs. 1 and 2) translocated through *E. coli* membrane and localized appreciably in the cytoplasm (Fig. 6). We had a very similar observation on the localization of these peptides onto *S. aureus* ATCC25923 also; NBD-IsCT localized predominantly onto the bacterial membrane whereas NBD-labeled versions of I9K-IsCT and E7K,I9K-IsCT were localized mostly onto bacterial cytoplasm (Fig. 6). The data clearly reveal a dissimilarity among these peptides in their localization onto bacteria and indicate the possibility of a difference in mode of action of IsCT and its non-toxic analogs I9K-IsCT and E7K,I9K-IsCT.

### Quenching of fluorescence of NBD-labeled peptides by trypan blue onto bacteria

Translocation of NBD-labeled peptide inside the bacteria was further probed by incubating the NBD-labeled peptide treated bacteria with trypan blue (TB). TB does not internalize into undamaged cells and therefore it can only quench the fluorescence of NBD-labeled peptides that are accessible extracellularly and has been used for phagocytosis studies^26^, translocation of cell penetrating peptides into eukaryotic cells^27^ uptake of peptides into bacterial cells^28^ etc. Bacteria were first treated with different NBD-labeled peptides, e.g. NBD-IsCT or NBD-E7K,I9K-IsCT followed by the addition of 1 mg/ml of TB with incubation for 10 min at room temperature and then analyzed by FACS. Fluorescence of *E. coli* treated with NBD labeled IsCT was quenched appreciably by TB probably indicating the localization of NBD-IsCT onto the membrane of *E. coli*. On the contrary, the fluorescence intensity of NBD-E7K,I9K-IsCT treated bacteria after the incubation with TB was quenched only slightly indicating that the NBD-labeled E7K,I9K-IsCT most probably translocated into the bacteria and therefore not accessible/quenched by TB (Supplementary Fig. S4). Thus TB quenching of fluorescence of NBD-labeled peptides bound to bacteria supports the confocal microscopic studies (Fig. 6).

### Assay of survival of bacteria in presence of IsCT and its analogs at shorter incubation period

**Figure 7:**
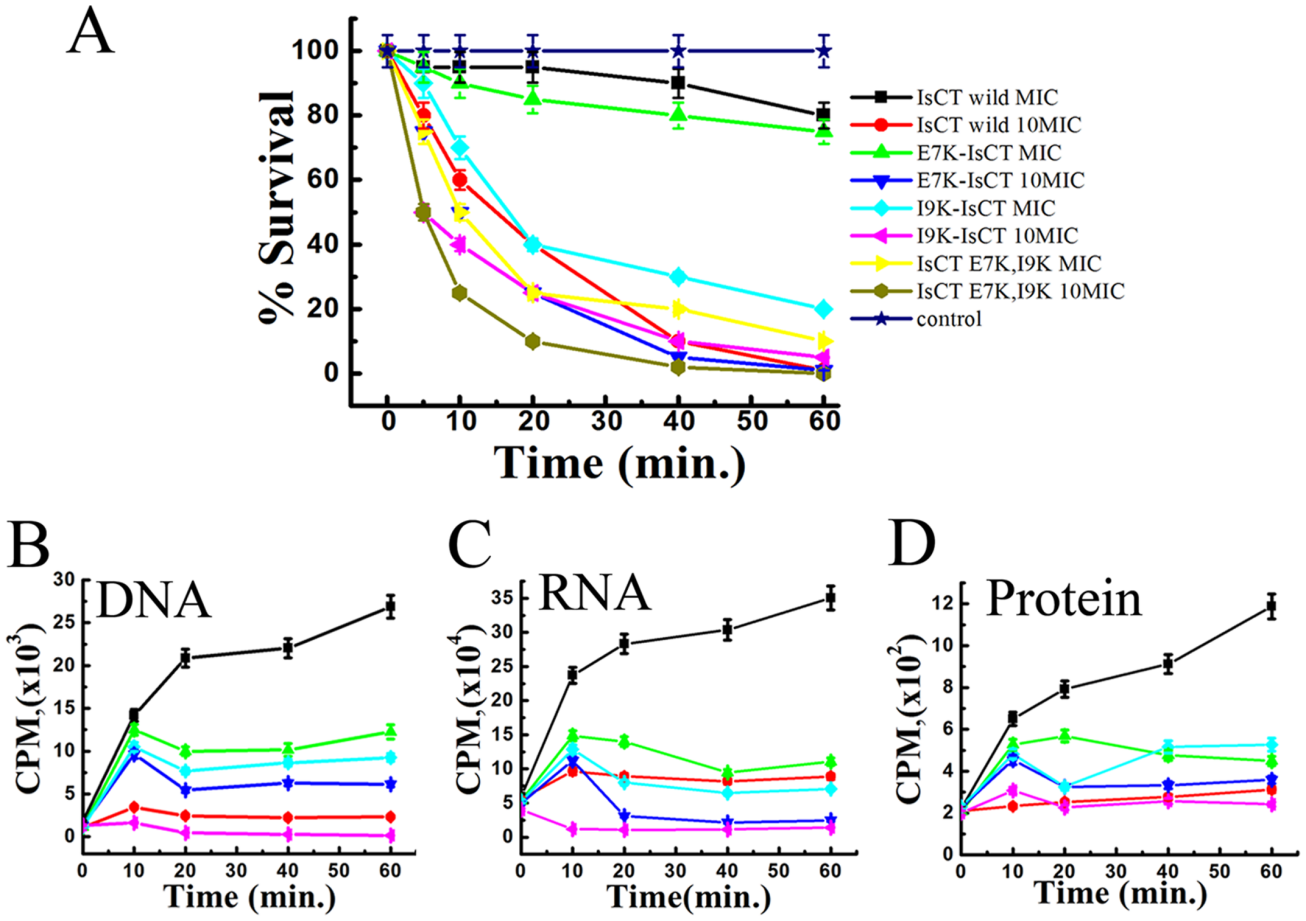
Survival assay of *E. coli* 25922 in the presence and absence of peptide and the effect of Peptides on macromolecular syntheses in *E. coli* ATCC 25922. (A), Bacterial survival assay; 10 μl samples were taken at the pointed out intervals and plated, incubated at 37°C overnight. Percent survival relative to the number of bacteria prior to peptide addition was determined. [^3^H] thymidine incorporation into DNA (B), [^3^H] uridine incorporation into RNA (C), and L-[^3^H] leucine incorporation into protein (D) were measured. The peptide I9K-IsCT was added at its MIC (upright triangle) and 10 times its MIC (inverted triangle), peptide I9K,E7K-IsCT was added at its MIC (diamond shape) and 10 times of MIC (triangle pointed left), Antibiotic as a positive control (ciprofloxacin 1 μg/ml for DNA, Rifampicin 2 μg/ml for RNA and Tetracycline 2 μg/ml for Protein synthesis inhibition) was added (circle). The results for control sample with no peptide are also shown (square). Each data point is an average of three independent experiments, and error bar represents the ± standard deviation.

Bacterial survival assays were performed at MIC and 10x MIC concentrations of IsCT and its analogs for 60 min incubation time with bacteria. Survival of *E. coli* was ~80% as a result of incubation with IsCT or E7K-IsCT at MIC (Fig. 7A). Interestingly, for the same incubation period with I9K-IsCT and E7K,I9K-IsCT respectively at their MIC, bacterial survival were 30% and 20% only. However, with incubations at 10x MIC peptide concentrations for IsCT and its analogs bacterial survival were 0–5%. Altogether, the data indicate a better efficacy of the non-toxic, IsCT-analogs, I9K-IsCT and E7K,I9K-IsCT in inhibiting the growth of bacteria at their MICs.

### Inhibition of macromolecular syntheses in E. coli ATCC25922 by IsCT-analogs, I9K-IsCT and E7K,I9K-IsCT

To explore if the cell-selective analogs of IsCT, I9K-IsCT and E7K,I9K-IsCT that do not permeabilize bacterial membrane despite significant anti-bacterial properties possess intracellular target and can stop syntheses of macromolecules in bacteria, the incorporation of radioactive precursors viz [methyl-3H] thymidine, [5-3H] uridine and L-[4,5-3H(N)] leucine into DNA, RNA and protein respectively was studied in presence of MIC and 10 fold MIC concentrations of these peptides in *E. coli*. A dose and time dependent inhibition of DNA synthesis by these peptides was observed (Fig. 7B). At MIC values I9K-IsCT and E7K,I9K-IsCT inhibited DNA synthesis after 20 min incubation with bacteria, whereas at 10 fold MIC value, I9K-IsCT inhibited DNA synthesis after 20 min of incubation and E7K,I9K-IsCT inhibited DNA synthesis in less than 10 minute of incubation (Fig. 7B). A similar pattern was observed in inhibition of RNA synthesis by these peptides (Fig. 7C). However, a delay was observed in inhibition of the protein synthesis as compared to the inhibition of DNA and RNA synthesis by these peptides (Fig. 7D). At MIC, inhibition of protein synthesis appeared after 40 minutes incubation of both the peptides whereas at 10× MIC peptide concentration both I9K-IsCT and E7K,I9K-IsCT significantly inhibited protein synthesis after 20 minutes of incubation (Fig. 7D). Altogether the results suggest an inhibition of nucleic acid and protein syntheses in *E. coli* ATCC25922 by I9K-IsCT and E7K,I9K-IsCT.

## Discussion

Significant reduction in cytotoxicity along with unprecedented alteration in mode of action of short antimicrobial peptide, IsCT was observed when an isoleucine residue, located in its hydrophobic face and also at an ‘a’ position of its identified leucine zipper sequence, was replaced (I9K-IsCT) with a cationic, lysine residue. The result is distinct from that of our previous studies where substitution of leucine/isoleucine residue at ‘a’/‘d’ position with alanine residue reduced the cytotoxicity of antimicrobial peptides but did not alter their mode of action against microorganisms^1^,^5^. An analog of IsCT, E7K-IsCT, was also characterized in which a glutamic acid residue located in the hydrophilic face of the peptide was replaced by a lysine residue. Combined impact of lysine substitutions in both hydrophobic and hydrophilic faces was studied by designing an analog, E7K,I9K-IsCT in which both the amino acid substitutions were made. E7K-IsCT was found to have less toxicity than IsCT; however cytotoxicity of I9K-IsCT and E7K,I9K-IsCT was insignificant against human RBCs as well as much lesser against murine 3T3 cells (Fig. 1A,1B). All the peptides showed potent antibacterial (1–8 μM) activity with their therapeutic index follow the order IsCT < E7K-IsCT < I9K-IsCT < E7K,I9K-IsCT.

I9K-IsCT and E7K,I9K-IsCT did not permeabilize the zwitterionic lipid vesicles or induced damage to mammalian cell membrane (Fig. 1C,1D) which is consistent with their non-cytotoxic nature. However, it was very interesting that despite having significant antibacterial properties, unlike IsCT and E7K-IsCT the other two analogs, I9K-IsCT and E7K,I9K-IsCT neither induced damage towards bacterial membrane nor permeabilized the bacterial membrane mimetic negatively charged lipid vesicles (Fig. 1D). This observation was a strong indication towards the non-membrane lytic mechanism of I9K-IsCT and E7K,I9K-IsCT against bacteria.

Considering the negligible shifts of tryptophan emission maxima of I9K-IsCT and E7K,I9K-IsCT in presence of bacterial membrane mimetic, negatively charged lipid vesicles (Fig. 3B), it was surprising to observe the poor acrylamide quenching of their fluorescence when the peptides were bound to this kind of vesicles (Fig. 3D) which suggest the non-accessibility of their tryptophan residues under such condition. However, from these two contrasting data on tryptophan emission maxima (Fig. 3B) and acrylamide quenching of tryptophan fluorescence of I9K-IsCT and E7K,I9K-IsCT in presence of negatively charged lipid vesicles (Fig. 3D) one could anticipate that tryptophan residues of these two peptides were most likely located in the inner space of this kind of bilayer membrane and hence not accessible to acrylamide and also experienced more polar environment. Yet, a significant acrylamide quenching of tryptophan fluorescence of I9K-IsCT and E7K,I9K-IsCT in the presence of zwitterionic lipid vesicles (Fig. 3C) clearly suggest that these two non-toxic IsCT analogs interact differently with zwitterionic and negatively charged lipids. Altogether, the tryptophan fluorescence and acrylamide quenching studies could be implicated to the translocation of two non-toxic IsCT analogs namely, I9K-IsCT and E7K,I9K-IsCT, inside the bacterial membrane mimetic negatively charged lipid vesicles.

Helical propensity of IsCT and E7K-IsCT in mammalian membrane mimetic, zwitterionic lipid vesicles matches with their cytotoxic nature. Likewise, insignificant helical structures of I9K-IsCT and E7K,I9K-IsCT in zwitterionic lipid vesicles (Fig. 4B) are supportive of their non-cytotoxic nature. Though secondary structure is not an essential requirement for their antimicrobial property^2^, many membrane-active antimicrobial peptides show significant helical propensities in bacterial membrane mimetic negatively charged lipid vesicles and there are reports on other form of secondary structures by these kinds of peptides as well^1^,^29^. Appreciable helical structures of IsCT and E7K-IsCT in negatively charged lipid vesicles (Fig. 4C) and permeabilization of this kind of model membrane (Fig. 1C,1D) match with each other and in fact both the peptides depolarize and damage the organization of bacterial membrane (Fig. 1C,1D) and can cause significant change in bacterial morphology (Fig. 5). On the contrary, despite having significant anti-bacterial activities, I9K-IsCT and E7K,I9K-IsCT practically showed no helical propensity in negatively charged lipid vesicles (Fig. 4C) and the peptides did not permeabilize this kind of lipid vesicles, depolarize and damage the organization of bacterial membrane (Fig. 1, 2) and also did not induce any significant alteration in bacterial morphology (Fig. 5). Interestingly, both I9K-IsCT and E7K,I9K-IsCT with no helical structure easily translocated through the bacterial membrane and mostly concentrated into its cytoplasm whereas helical and cytotoxic IsCT localized mostly onto the bacterial membrane as evident by confocal microscopy (Fig. 6) and FACS studies (supplementary Fig. S4). Thus the present study provides ample evidence in favor of the possible membrane-lytic mechanism of IsCT or its partly cytotoxic analog, E7K-IsCT. The studies with radioactive isotopes clearly showed that IsCT analogs, I9K-IsCT and E7K,I9K-IsCT inhibit nucleic acids and protein syntheses in tested *E. coli* (Fig. 7). Thus the data explain the mode of action of I9K-IsCT and E7K,I9K-IsCT that translocate into the bacterial cytoplasm without permeabilizing its membrane. Though majority of the antimicrobial peptides show their antimicrobial property by lysis of bacterial membrane there are a few antimicrobial peptides including histatins, pyrrhocoricin, drosocin, apidaecin and cathelicidin-derived peptides bactenecin, PR-39 and prophenin and tryptophan and proline rich bovine antimicrobial peptide, indolicidin that inhibit biomolecular syntheses as their mechanism of action^19^,^30^,^31^,^32^,^33^,^34^,^35^,^36^,^37^.

The novel IsCT analogs, I9K-IsCT and E7K,I9K-IsCT being reported here add to the list of only few peptides^10^,^13^,^16^,^38^ that possess intracellular target. Buforin II, identified in Asian toad was shown to stop biomolecular syntheses in bacteria and it was demonstrated that the proline hinge present in this molecule plays an essential role for its cellular penetration^39^. Though, it is not clear how the proline hinge is crucial for the cellular penetration of buforin II, it seems that the structural flexibility caused by the distortion in helical structure made by the proline hinge could be important for the efficient translocation of the peptide^39^. We speculate that the deformation in helical structure as a result of substitution of isoleucine residue at 9^th^ position with a lysine residue (Fig. 4C) may provide similar flexibility to IsCT for its translocation through the bacterial membrane to cytoplasm. Not only in IsCT, the same amino acid substitution converted helical and membrane-active, E7K-IsCT into a random coil (Fig. 4C), cell-penetrating peptide that can inhibit macromolecular syntheses in *E. coli*.

What is striking about these IsCT analogs, I9K-IsCT and E7K,I9K-IsCT is that these peptides seem to translocate only to the bacterial membrane; no penetration of fluorescent versions of these peptides through hRBC membrane was evident from confocal microscopic studies (Fig. 6). A single isoleucine to lysine substitution at an ‘a’ position of the leucine zipper sequence of IsCT resulted in a remarkable change in secondary structure (Fig. 4C) from helical to random coil in bacterial membrane mimetic environment and also transformed a predominantly membrane active molecule into a membrane translocating molecule. To our knowledge there is no report in the literature where a point mutation in the leucine zipper sequence of an antimicrobial peptide results in such alteration of secondary structure in bacterial membrane mimetic environment and its mode of action. However, it has been mentioned in a very recent article^40^ that when polar or charged residues occupy the ‘a’ and ‘d’ positions of leucine zipper coiled coil sequence, it leads to destabilization of the coiled coil motif. On the basis of analyses on motor and structural proteins by mostly computational approaches^41^,^42^ it has been predicted that as a result of such substitutions the polypeptide will either exist as a monomeric random coil or will form single α-helical domain^40^. Both I9K-IsCT and E7K,I9K-IsCT do not contain a significantly high number (6 or more) of positively charged residues like HIV Tat^43^ or penetratin^44^ or Hsp70^45^,^46^. While I9K-IsCT contains two net positive charges; the other analog, E7K,I9K-IsCT contains four net positive charges. These two IsCT analogs are not very hydrophobic either like cell penetrating protein signal sequences^47^; rather these peptides are amphipathic in nature without any proline hinge like that in buforin-II^39^, bacteriocins^48^, abaecin^49^, apidaecin^50^,^51^, IsCT-*P*^23^ *etc.* Thus the primary structures of these two IsCT analogs to our knowledge seem to represent new prototype amino acid sequences that possess cell-penetrating, non-membrane lytic and intracellular targeting properties in *E. coli*.

To summarize, the results presented here clearly suggest that isoleucine to lysine substitution at the hydrophobic face of IsCT which is also an ‘a’ position of its heptad repeat sequence led to its translocation through the bacterial membrane indicating its essential role in the translocation process of the peptide; however, the replacement of glutamic acid residue with a lysine residue in the hydrophilic face of the molecule could not result the same effect in IsCT implicating probably the importance of an amino acid at specific position. The results could aid in the design of novel antimicrobial agents of high therapeutic index and show a new approach for the design of cell-selective and cell-penetrating antimicrobial peptides. Considering that there is no methodology for the design of bacterial membrane penetrating small peptides with intracellular target, the present study could be very valuable. Since leucine/isoleucine or phenylalanine zipper sequences occur frequently in naturally occurring antimicrobial peptides probably a similar approach may be attempted for designing cell-selective, non-membrane lytic and intracellular-targeting analogs of these peptides.

## Experimental

### Materials

Rink amide MBHA resin (loading capacity: 0.4–0.8 mmol/g) and all the N-α -Fmoc and necessary side-chain protected amino acids were purchased from Novabiochem, Switzerland. Coupling reagents were purchased from Sigma, India and solvents for peptide synthesis and peptide purification by HPLC were purchase from Sigma, India or Merck, India. FITC-annexinV, valinomycin, and cholesterol (Chol) were purchased from Sigma. Egg phosphatidylcholine (PC) and egg phosphatidylglycerol (PG) were obtained from Northern Lipids Inc., Canada while 3,3′-dipropylthiadicarbocyanine iodide (diS-C_3_-5), NBD-fluoride (4-fluoro-7-nitrobenz-2-oxa-1, 3-diazole) and tetramethylrhodamine succinimidyl ester were procured from Invitrogen, India.

### Methods

#### Peptide synthesis, fluorescent labeling, and purification

The peptides were synthesized by PS-3 Protein Technologies automated synthesizer via the solid phase method on Rink amide MBHA resin utilizing standard F-moc chemistry [20]. Labeling of the peptides at their N-terminus with fluorescent probes like NBD and rhodamine, cleavage of the labeled and unlabeled peptides from the resin, peptide precipitation and their purification by reverse phase HPLC with a C18 column using a linear gradient of 20–80% acetonitrile for 40 min were carried out by a standard procedure as reported earlier [20]. The purity of the peptides was further determined to be ≥95 by reverse phase analytical chromatography. Molecular mass of the peptides were confirmed by MALDI-TOF.

#### Assay of hemolytic activity of the peptides

Fresh human blood, collected in the presence of an anti-coagulant, was kindly provided by Dr. S. K. Puri who is leading a malaria research group at our Institute and possesses an ethical approval from the Institutional ethics committee (CDRI/IEC/CEM/21-07-2010) for collecting human blood for research purpose. Human red blood cells (hRBCs) were separated from plasma by centrifugation followed by the washing with PBS for three times. Hemolytic activity of the peptides against hRBCs in PBS was performed as reported earlier^1^,^52^.

#### Antibacterial activity assay of the peptides

This bioassay with the peptides was done in 96-well microtiter plates against different Gram-positive bacteria and Gram-negative bacteria as reported before [21]. In brief, bacterial cultures were grown at 37°C in shaking incubator at 180 rpm in Mueller Hinton Broth medium in aerobic condition to the mid-log phase as determined by the optical density at 600 nm, which was 0.4 to 0.5, subsequently diluted in same media, then 50 μl bacterial culture (~10^6^ cfu/ml) were added to 50 μl of water containing two fold serially diluted different peptides in each well and incubated for 14–18 hours at 37**°**C. The peptide's antibacterial activities, expressed as their MICs (the peptide concentration which results 100% inhibition of microbial growth), were assessed by measuring the absorbance at 600 nm.

#### Computation of physicochemical parameters of the peptides

Basic hydrophobicity parameters such as mean hydrophobicity, hydrophobic moment and mean relative hydrophobicity moment were calculated by the software HydroMCalc on the basis of combined consensus hydrophobicity scale. The peptide's net hydrophobic mean characters were calculated by the GRAVY scale^53^. Further physicochemical properties such as aliphatic index were calculated by ExPASy algorithms.

#### Circular dichroism (CD) studies

The circular dichroism (CD) spectra of the peptides were recorded on Jasco J-815 spectropolarimeter in phosphate buffered saline (PBS, pH 7.4), zwitterionic PC/Chol (8:1, w/w) and negatively charged PC/PG (3:1 w/w) lipid vesicles with as usual procedure^1^,^24^. For secondary structure estimation, data analyzed by-JASCO CD, J-815, SSE (Secondary Structure Estimation) software.

#### Detection of peptide-induced membrane damage of hRBCs and bacterial cells

Peptide-induced phospholipid asymmetry or damage of phospholipid membrane organization of hRBCs was determined by staining the cells (~3.0 × 10^7^ cells/ml) with FITC-annexin-V^54^ after the treatment with the peptides at room temperature for 5 min. Extent of staining was measured by analyzing peptide treated cells with respect to peptide untreated control using Becton Dickinson FACS Calibur flow cytometer and CellQuest Pro software. Further, the probing of peptide-induced damage to membrane integrity of bacteria by propidium iodide staining was carried out and analyzed by the same flow cytometer as described elsewhere^24^.

#### Assay of peptide induced dissipation of diffusion potential

Peptides induced permeabilization of phospholipid membrane was measured by their ability to dissipate the diffusion potential across the lipid vesicles composed of either zwitterionic PC/Chol (8:1, w/w) and negatively charged PC/PG (3:1 w/w) lipid vesicles with the help of a potential sensitive dye diS-C_3_-5^1^,^24^.

#### Recording of tryptophan emission spectra quenching of its emission by acrylamide

Tryptophan fluorescence spectra of IsCT and its analogs were recorded in PBS and in the presence of small unilamellar vesicles (SUVs) composed of either PC/Chol (8:1, w/w) or PC/PG (3:1, w/w) with excitation for tryptophan at 280 nm and emission range of 300 to 400 nm as described earlier. The experiments dealing with quenching of tryptophan fluorescence by acrylamide were performed as reported earlier. The data were analyzed according to the Stern-Volmer equation, and *Ksv* is the Stern-Volmer quenching constant was determined as before^24^.

#### Determination of inhibition of biomolecular synthesis

The effects of IsCT peptide and its designed anologs on *E. coli* DNA, RNA and protein synthesis were studied as functions of incorporation of the radioactive precursors [methyl-3H]thymidine, [5-3H]uridine and L-[4,5-3H(N)]leucine respectively as described^55^,^56^. 1 × 10^6^ mid-log phase *E. coli* ATCC 25922 resuspended in 10 mM sodium phosphate buffer (pH 7.4) and were treated with MIC or 10 times MIC of IsCT wild type peptide and analogs and 5 μl/ml of either [methyl-3H]thymidine (20 Ci/mmol), [5-3H]uridine (25.5 Ci/mmol) or L-[4,5-3H(N)]leucine (59.5 Ci/mmol) for different time periods. After incubation at 37°C, bacterial suspension were added to 10% ice-cold trichloroacetic acid and allowed to stand in ice for 40 min. Samples were then collected on 2.5 cm GF/C glass microfiber filters (Whatsman) using vacuum filtration and washed thoroughly with 5% ice cold TCA and 70% ice cold ethanol. The filters were then dried and placed in scintillation vials containing 5 ml of EcoScint scintillation cocktail (MP Biosciences) and counts were obtained in a liquid scintillation counter (Perkin Elmer) for 1 min for each filter. Statistical analyses using Student's t-test were performed using Prism 3.0 software. Values shown are Mean ± S.D.

#### Scanning electron microscopy (SEM)

The morphological changes induced by peptides on *E. coli* were studied using scanning electron microscopy. Cells were fixed with 2.5% glutaraldehyde in phosphate buffer (pH 7.2). Samples were post-fixed in 1% osmium tetroxide and subsequently dehydrated through an ascending ethanol series, critical point dried and coated with Au-Pd (80:20) using a Polaron E5000 sputter coater. Bacterial morphology was examined in a FEI Quanta 250 using a SE detector at an accelerating voltage of 30 kV. At least 400 cells were analyzed for each sample from two independent experiments^56^.

#### Localization of peptides onto mammalian and bacterial membranes

Localization and binding of the peptides onto hRBCs, *S. aureus* and *E. coli* was studied using Rho- and NBD-labeled peptides respectively by employing a Carl Zeiss LSM 510 META (Carl Zeiss, Jena, Germany) confocal laser scanning microscope as described earlier. The confocal microscope is equipped with 405 nm diode, Argon multiline (458, 477, 488, 514 nm), 561 nm DPSS and a HeNe 633 nm lasers. Images were analyzed using the Zeiss AIM ver 4.2 software.

#### Cell viability assay

Viability of the cells were determined to check the toxic activity of peptides against murine 3T3 by a standard MTT assay as described earlier^57^. Readings of these samples were taken at 550 nm by ELISA reader^57^. The viability of the peptide-treated cells was calculated with respect to the control cells of 100% viability.

## Author Contributions

J.K.G. conceived the idea. J.K.T. performed majority of the experiments. Confocal microscopic and SEM experiments were performed by M.K. which was supervised by K.M.; K.M. also prepared these images for the manuscript. A.K. assisted J.K.T. in peptide syntheses. J.K.T. and J.K.G. analyzed the data and wrote the manuscript. All the authors were consulted on preparation of the manuscript; J.K.G. arranged funding for this work.

## Supplementary Material

Supplementary Information Supplementary information

## Figures and Tables

**Table 1:** Designations and amino acid sequences of native IsCT and its designed analogs

Peptide		Sequence	Calculated	Observed
			mass	Mass
		g**a**bc**d**efg**a**bc**d**e		
IsCT		X-NH-I**L**GK**I**WEG**I**KS**L**F-CONH_2_	1503.97	1504.0
E7K-IsCT		X-NH-I**L**GK**I**W**K**G**I**KS**L**F-CONH_2_	1503.03	1502.7
I9K-IsCT		X-NH-I**L**GK**I**WEG**K**KS**L**F-CONH_2_	1518.99	1518.3
E7K,I9K-IsCT		X-NH-I**L**GK**I**W**K**G**K**KS**L**F-CONH_2_	1518.05	1518.0

*Amino acids at ‘a’ and ‘d’ positions of the identified leucine zipper sequence are in bold letters while the substituted amino acids are marked as bold and underlined.

**Table 2:** Antibacterial activity of IsCT and its designed analogs

Bacterial strains	Minimum inhibitory conc. (MIC)^a^ in μM			
	IsCT peptide and analogs			
	IsCT	E7K	I9K	E7K,I9K
**Gram positive strain**				
*S. aureus* (ATCC 25923)	2 ± 0.3	2 ± 0.2	8 ± 0.5	1 ± 0.2
*B.substilis*(ATCC 6633)	3 ± 0.4	2 ± 0.3	2 ± 0.3	1 ± 0.2
**Gram negative strain**				
*E.coli* (ATCC 25922)	6 ± 0.5	4 ± 0.4	8 ± 0.5	2 ± 0.3
*E.coli* (ATCC 10536)	3 ± 0.3	2 ± 0.3	4 ± 0.3	1 ± 0.2
*P. aeruginosa*(ATCC BAA-427)	4 ± 0.5	3 ± 0.4	8 ± 0.6	2 ± 0.3

^a^MICs were determined from the mean of values obtained from three independent experiments, each performed in duplicate ± the standard deviation.

**Table 3:** Therapeutic index of IsCT wild type and its designed novel analogs

	HC_50_(μg/ml)	MIC(μg/ml)	Therapeutic index#
Peptides	hRBCs	*E. coli*	(HC_50_/MIC)
IsCT	43.59	9.02	4.83
E7K-IsCT	92.17	6.01	15.34
I9K-IsCT	500*	12.02	41.60
E7K,I9K-IsCT	500*	3.01	166.11

^#^Therapeutic index = HC50/MIC, where HC50 value is the mean concentration of peptide producing 50% hemolysis of fresh human erythrocytes donated by a healthy donor, while MIC is defined as the minimum inhibitory concentration using the microdilution susceptibility test against *Escherichia coli*. *When no detectable hemolytic activity was observed at 250 μg/ml, a value of 500 μg/ml was used for calculation of the therapeutic index. Larger values indicate greater antimicrobial specificity.
