# Supplementary material for: An Unprecedented alteration in mode of action of IsCT resulting its translocation into bacterial cytoplasm and inhibition of macromolecular syntheses
Source: Sci Rep. 2015 Mar 16;5:9127. doi: 10.1038/srep09127 (PMC4360471; doi:10.1038/srep09127)
Supplement: Supplementary Information [file srep09127-s1.pdf]

Supplementary information

An Unprecedented alteration in mode of action of IsCT resulting its translocation into bacterial cytoplasm and inhibition of macromolecular syntheses

Jitendra K. Tripathi[1], Manoj Kathuria[2], Amit Kumar[1], Kalyan Mitra[2] and Jimut K. Ghosh[1]#

[1]Molecular and Structural Biology Division

[2]Electron Microscopy Unit

CSIR-Central Drug Research Institute,

Sector 10, Jankipuram Extension, Sitapur Road

Lucknow–226 031, India

[#]To whom correspondence should be addressed: Jimut K. Ghosh, Molecular and Structural Biology Division, CSIR-Central Drug Research Institute, Sector 10, Jankipuram Extension, Sitapur Road, Lucknow–226 031, India,Tel: 091-522-2771940 (Ext.-4451); Fax: 091-522-2771941, E-mail: jighosh@yahoo.com; jk_ghosh@cdri.res.in.

Supplemental Tables

Supplementary Table S1: Physicochemical parameters of IsCT and its analogs

Peptides	μ−rel H (Mean relative H moment)	μH (Mean H moment)	<H> (Mean Hydrophobicity)	Aliphatic Index	GRAVY
IsCT	0.81	5.13	2.15	150.00	0.777
E7K-IsCT	0.83	5.25	2.03	150.00	0.746
I9K-IsCT	0.59	3.71	0.72	120.00	0.131
E7K,I9K-IsCT	0.61	3.83	0.59	120.00	0.100

Supplementary Table S2: Secondary Structure Estimation (SSE)* of IsCT and its analogs

Peptide	% Helix	% Beta	% Turn	% Random
**A. SSE of peptides in PBS (pH 7.4)**				
IsCT	0	39.0	0.0	61.0
E7K- IsCT	0	32.7	7.3	60.0
I9K- IsCT	0	25.5	3.0	71.5
E7K,I9K- IsCT	0	27.0	5.7	67.3
**B. SSE of peptides in presence of PC/Chol lipid vesicles**				
IsCT	28.3	20.8	18.0	32.9
E7K- IsCT	15.3	30.1	13.9	40.7
I9K- IsCT	0.0	24.8	5.3	69.9
E7K,I9K- IsCT	0.0	25.5	10.7	64.0
**C. SSE of peptides in presence of PC/PG lipid vesicles**				
IsCT	29.2	21.2	17.8	31.0
E7K- IsCT	25.9	20.1	19.7	34.3
I9K- IsCT	0.0	32.2	4.5	63.2
E7K,I9K- IsCT	0.0	24.7	8.7	66.6

*Data analyzed by-JASCO CD, Model Name-J-815, SSE (Secondary Structure Estimation) software.

Supplemental Figure:

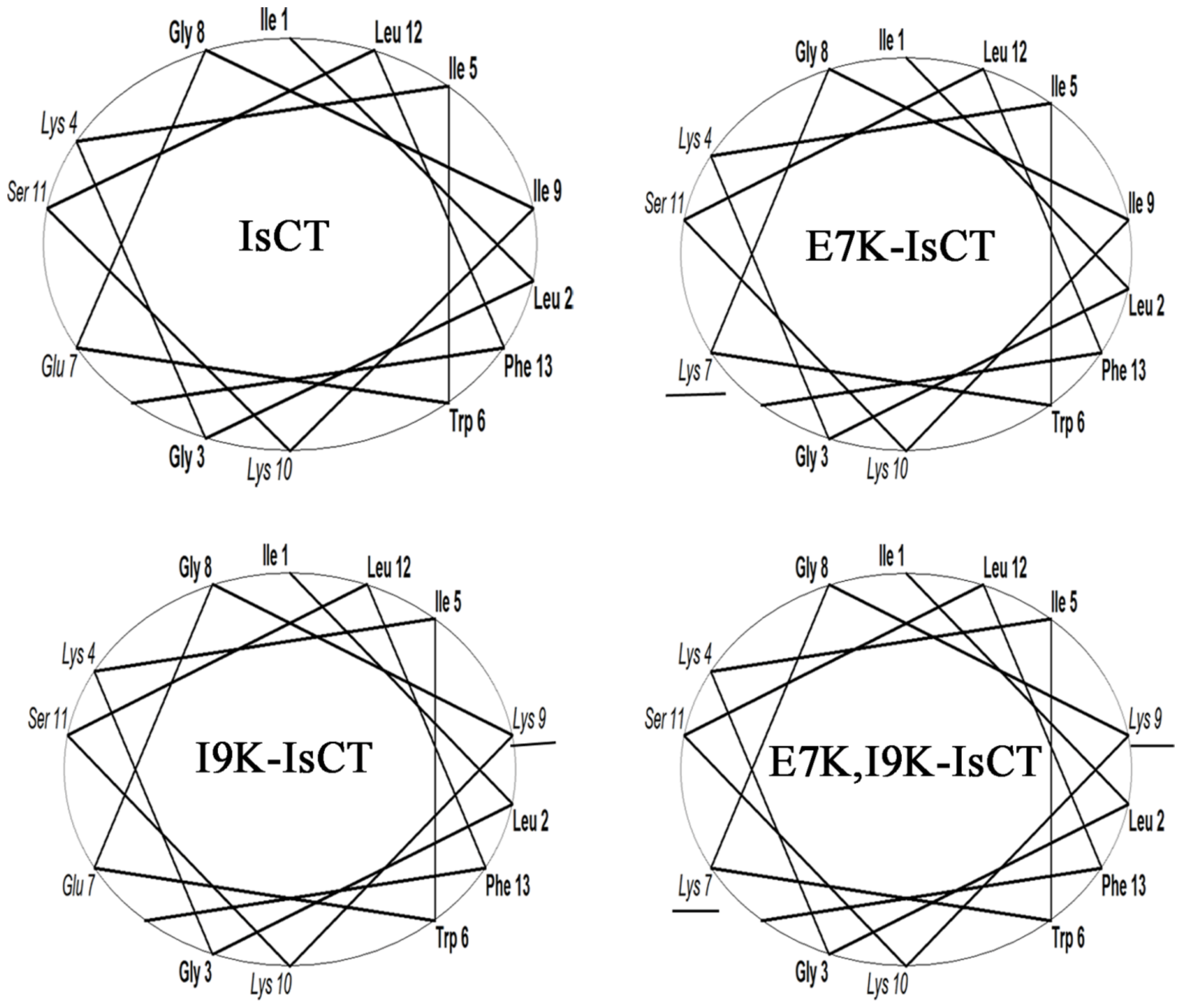

Supplementary Figure S1: Helical wheel projections of IsCT and its analogues. Substituted amino acids are underlined

Figure S2

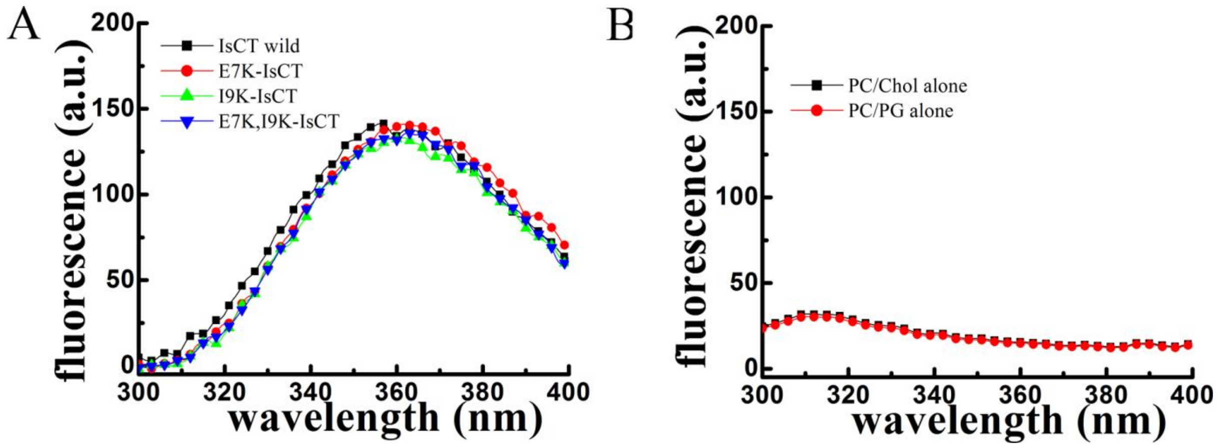

Supplementary Figure S2: Intrinsic Tryptophan fluorescence of peptides alone (A) and of PC/Chol and PC/PG alone (B), in PBS. Concentration of the each peptides used was ~2.0 μM and lipid concentration was ~450 μM.

Figure S3

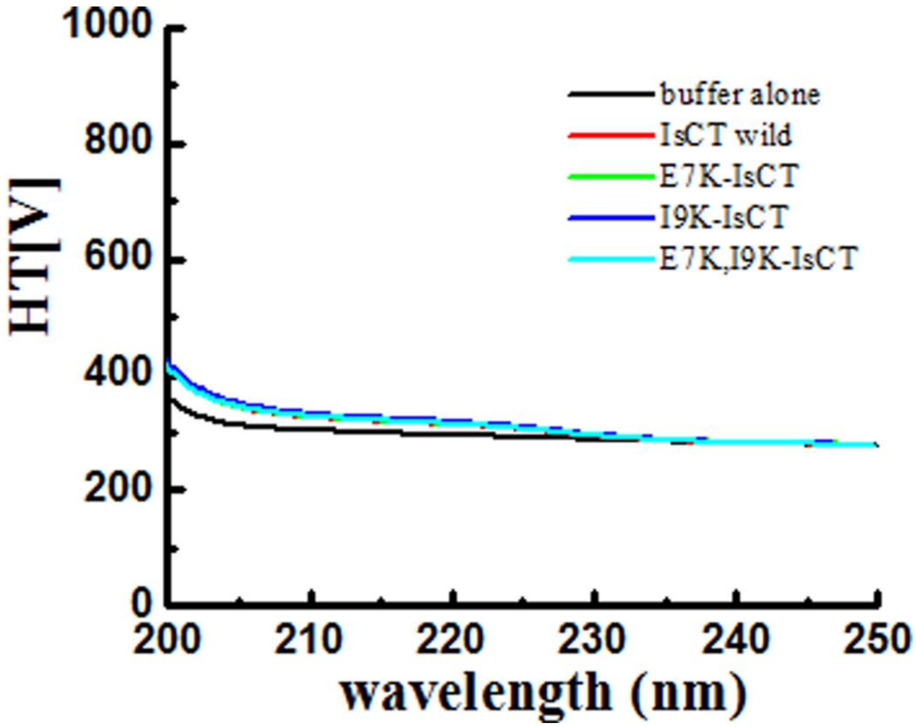

Supplementary Figure S3: Change in HT voltage of the buffer as well different peptides in PBS, pH 7.4. Concentration of the each peptides used was ~43 μM.

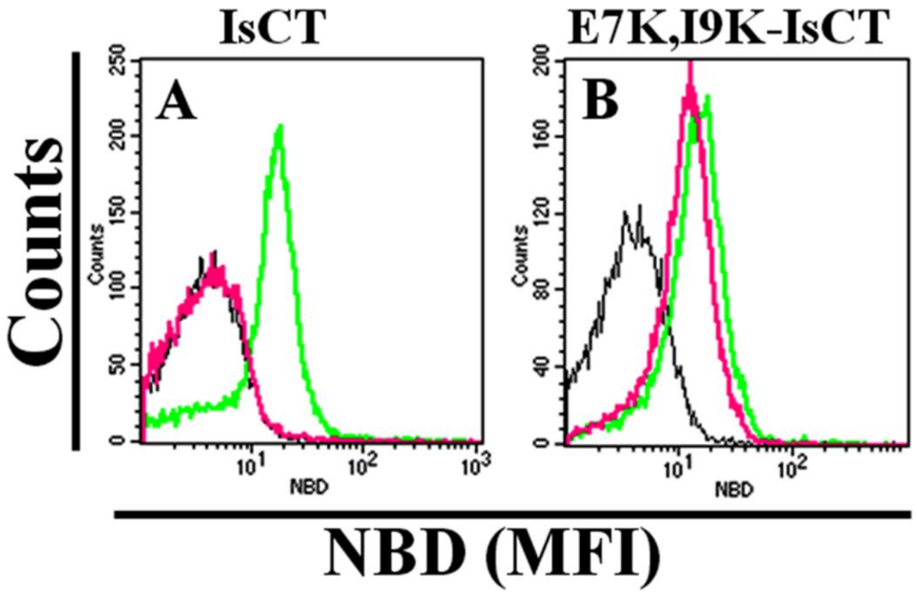

Supplementary Figure S4: Quenching of NBD fluorescence of *E. coli ATCC 25922* bacteria bound to NBD-labeled IsCT or E7K,I9K-IsCT as marked in the Fig. by trypan blue. *E. coli* (1x10^6 CFU/ml) were incubated with or without NBD-labeled peptides (either NBD-labeled IsCT or E7K,I9K-IsCT at 2.0μM peptide concentration), washed, and analyzed by flow cytometry; Symbols: with NBD-labeled peptide, green histograms and without peptide, black histograms. *E.coli* bacteria treated with each of the NBD-labeled peptides were separately incubated with Trypan Blue (1mg/ml) for 10 min at 37°C and further analyzed by flow cytometry (pink histograms).
